# “An invitation to think differently”: a narrative medicine intervention using books and films to stimulate medical students’ reflection and patient-centeredness

**DOI:** 10.1186/s12909-023-04492-x

**Published:** 2023-08-11

**Authors:** Elsemarijn Leijenaar, Charlotte Eijkelboom, Megan Milota

**Affiliations:** 1 Julius Center for Health Sciences and Primary Care, University Medical Center Utrecht, Utrecht University, Utrecht, The Netherlands; 2 Department of Pediatrics, University Medical Center Utrecht, Utrecht University, Utrecht, The Netherlands

**Keywords:** Narrative medicine, Reflective writing, Patient-centeredness, Medical students

## Abstract

**Background:**

Narrative medicine purports to improve medical students’ communication skills, self-reflection, empathy and professional development. These interpersonal skills and attitudes can facilitate more patient-centered care and positively impact patients’ treatment outcomes. However, current studies report mainly on small study populations, leaving the efficacy of narrative medicine in larger study groups unknown. Therefore, this study aimed to evaluate a mandatory narrative medicine lesson in a large sample of medical students. We assessed if this narrative medicine lesson stimulated meaningful self-reflection on patient-centeredness in medical students.

**Methods:**

All fourth-year medical students of a Dutch medical school participated in this mandatory narrative medicine lesson. The four-step activity consisted of an introductory lecture, close reading and watching of a book and film, a discussion group and a short reflective writing exercise. Students were divided over three thematic pairings (or book and film combinations): ‘The doctor as a patient,’ ‘The mysterious brain,’ and ‘Until death do us part.’ A mixed-methods design was used. First, we qualitatively analyzed the content of 203 essays from the reflective writing exercise. A quantitative analysis of the reflective quality was conducted using a scoring-system based on the REFLECT rubric.

**Results:**

Students demonstrated reflection on a professional level and connected this to future intentions as medical practitioners, for example to use specific communication strategies or to deliver healthcare in a broader sense. They also reflected on a personal level by questioning their own worldview or beliefs. Furthermore, they stressed the importance of individual patient stories to gain understaning of patients’ perspectives. Approximately half of the students showed an in-depth and authentic reflection, according to the REFLECT rubric. Additionally, reflection levels differed between book and film pairings.

**Conclusions:**

This study supports the value of narrative medicine in the medical curriculum by validating its outcomes in a larger study population and in a mandatory course. Students reflected on themes related to the principles of patient-centeredness, namely personal attitudes towards patients and the uniqueness of patient stories. In additon, the majority of students demonstrated higher levels of reflection, which suggests that this exercise contributes to self-awareness and self-reflection, important qualities for delivering patient-centered healthcare.

## Background

There is an increased recognition of the importance of patient preferences, the social and cultural contexts of care, and patients’ individual life stories [1]. Indeed, patients who feel like active participants in their care trajectories via patient-centered care approaches, like shared-decision making, also experience better health outcomes [2, 3]. Scholl et al. systematically mapped the concept of patient-centeredness and identified underlying factors that can optimize patient-centered care [4, 5]. These factors include investing in the clinician-patient relationship, acknowledging the uniqueness of each patient’s lived experiences, and acknowledging biopsychosocial factors. Clinicians’ interpersonal skills, such as empathy, compassion, trustworthiness and self-reflectiveness, can also contribute to patient-centered care [4, 5]. Sandars [6] adds that clinicians’ awareness of their own underlying beliefs and values can positively impact the clinician-patient relationship. This relationship between clinicians’ self-awareness and empathic healthcare has also been studied by Dasgupta et al. [7] using reflective writing. As they quote in their study: *“It takes a whole doctor to treat a whole patient”*[8]. Reflective writing is a powerful tool that has been extensively used to promote (future) clinicians’ professional development, self-understanding and sense of connection to their colleagues and patient communities [9, 10].

However, stimulating the development of medical students’ sense of empathy, compassion and self-awareness remains challenging. Veen et al. [11] describe these attitudes as private experiences, which are difficult to train or articulate in classroom settings. In another article, they note that requiring students to map or assess their own development in interpersonal skills (such as empathy, compassion and self-awareness) via required writing assignments runs the risk of eliciting inauthentic or ‘zombie-like’ self-reflections. This is further exacerbated by educators’ attempts to transform highly individualized and varied interpersonal skills into uniform, measurable learning outcomes [12].

Narrative medicine (NM) was founded at Columbia University and has been proposed as a teaching model for patient-centered medical practice by fostering attentive listening and clinician-patient affiliation [1, 10, 13]. Narrative competence, according to the NM founders, constitutes *“the ability to acknowledge, absorb, interpret and act on the stories and plights of others” *[13]. NM as a pedagogic strategy employs different art forms, close reading exercises and creative writing [14]. It provides students with a broader perspective on the experience of illness, which they can use in their interactions with patients. It also helps students better understand their own life journey, which in turn can help them recognise their own feelings and emotions during patient interactions and can eventually lead to more authentic engagements with patients [13].

So far, multiple studies have described the beneficial effects of NM as a pedagogical tool for medical students, with a positive impact on communication skills, empathy, self-reflection, and relationship-building [14–16]. However, most of these studies report on small study groups predominantly in elective courses, which leaves the efficacy of NM in larger study groups largely unknown [17–26]. The aim of this study was to explore if a mandatory narrative medicine lesson could stimulate meaningful self-reflection and (themes related to) patient-centeredness in medical students.

## Method

### Setting

In this mandatory exercise, students read a book, watched a film and discussed these art forms in small groups (Table 1). First, during the preparatory lecture, students received a brief introduction about the main tenants and goals of NM. Second, students were assigned to one of three book and film pairings*.* Each pairing related to one of the specialties of their current clerckships (neurology, psychiatry and geriatrics). The pairing ‘The doctor as a patient’ concerned the illness experience of physicans getting a life-changing neurological disease, the pairing ‘The mysterious brain’ concerned the lived experience of persons with autism spectrum disorder and their relatives, and the pairing ‘Until death do us part’ concerned the experiences of partners of elderly patients during the last phase of their lives. Students were asked to read the book and watch the film individually during a span of 4 weeks. As preparation for the follow-up group discussion, they were asked to select a fragment from the book or the film that impacted them and to compile their own questions and comments to share with the group during the discussion. Finally, during the 45-min small group discussion (max. 6 students), students discussed the art forms under the guidance of faculty members from the department of Global Public Health, Bioethics and Health Humanities. At the end of the session, students completed a written reflection exercise and shared their answers with the group; written responses were collected by faculty members at the end of the lessons.

**Table 1:** Description of narrative medicine lesson

**Goal**			
To provide care that is better aligned with the expectations and wishes of patients			
**Content**			
**Theme**	**Book**	**Film**	
The doctor as a patient	Paul Kalanithi, *‘When breath becomes air’*	Médecin de Campagne (2018)	
The mysterious brain	Mark Haddon, *‘The curious incident of the dog in the night-time’*	Life, Animated (2016)	
Until death do us part	Alice Munro, *‘The bear came over the mountain’*	Amour (2012)	
**Outline**			**Time (min)**
**Week 1: Preparatory lecture** **Week 1–6: Students read book, watch film** **Week 6: Small group discussion**			
*Introduction:*			
- General Introduction by faculty member and explanation of the learning goals			5
*Student discussion:*			
- Student-led discussion, guided by the faculty member when necessary			25–30
*Reflective writing:*			
- Each student had to write a short essay with answers to the following questions: ◦ What did you appreciate about this lesson? ◦ What recommendations do you have to improve this lesson? ◦ Write a short reflection where you incorporate the following questions: ‘What did this book/film teach you about the doctor-patient relationship? What lesson have you learned that you can apply in your professional career?’			10
- 1 or 2 volunteers share their response followed by a brief discussion			5

Faculty members all attended an instruction session led by one of the co-authors (M.M.) and were given a teacher manual with theoretical background information about the lesson, sample discussion questions, and the suggested lesson structure (Table 1).

### Study participants and lesson context

All fourth-year medical students at the University Medical Center Utrecht (UMCU) who attended the NM lesson between October 2018 and March 2020 were included in this study. Exclusion criteria were: students whose handwritten reflections were not readable, students who did not complete the written reflection exercise or essays that were not submitted to the lesson coordinator by the teacher.

The NM lesson is a mandatory part of the longitudinal ‘Patient Perspectives Program’ of the UMCU medical curriculum and takes place during students’ longitudinal clerkship of neurology, psychiatry and geriatrics.

### Data analysis

A mixed-methods design was used analyze students’ written reflections because we wanted to study the content as well as the quality of the reflections. First, essays were thematically analyzed in NVivo 12 using an inductive approach [27]. Two researchers (E.L. and M.M.) independently coded a sample of 25 essays of each book and film pairing for recurrent themes and concepts. Codes and themes were compared, discussed and combined to form the preliminary codebook. Subsequently, both researchers applied this codebook to 10 essays from each of the three pairings. They discussed their findings and revised and added new codes. After reaching agreement the first author (E.L.) used the final codebook to code all essays. Students’ feedback and recommendations were included in the thematic analysis to get insight into the lesson experience as well.

Second, a quantitative analysis was conducted to assess the level of students’ reflections using a scoring-system based on ‘The Reflection Evaluation For Learners’ Enhanced Competencies Tool’ (REFLECT) [28]. The REFLECT rubric was originially designed for formative assessment, but was chosen for this study as a means of gaining a more objective sense of the quality of students’ reflection. For this study, a simplified version of the REFLECT rubric was created; essays were scored in their entirety on a scale from 1-4 instead of scoring each criterion in the original REFLECT rubric (Table 3, criteria). This adaptation was necessary because we evaluated short reflections, whereas the REFLECT rubric is designed to assess longer reflections. The scores 1-4 respectively represent habitual action (1), thoughtful action or introspection (2), reflection (3) and critical reflection (4).

All authors assessed the level of reflection in the student essays using the simplified REFLECT rubric. To reach consensus about the scoring system, all authors scored a sample of 25 essays from each of the three pairings. Differences in scoring were discussed and the scoring system was refined. All authors then scored an additional sample of 10 essays for each pairing. After meeting again to discuss differences in scores, the scoring system was finalized. Then, the authors independently scored all 203 essays and the two-way random-effects intraclass correlation coefficient model was performed to assess the agreement between the three raters, [29]. Analysis showed that there was a good agreement between the authors with an intraclass correlation of 0.96 (95%CI 0.94-0.97), Where authors scored essays differently, the median score was used as final score. The data was not normally distributed, therefore the median reflection scores were used. To compare scores between the book-film pairings, we used the Kruskall Wallis test to calculate the p-value (since our data was not normally distributed and ordinal). The p-value demonstrates the probability that differences in scores between the book-film pairings were based on coincidence. SPSS 27.0 was used to conduct these quantitative analyses.

## Results

345 students followed the NM exercise. Students were excluded because their handwritten reflection was unreadable, because they had not written or submitted a reflection, or the reflections had been misplaced by the teacher (n = 136). This left 203 students’ essays from the reflective writing exercise (‘The doctor as a patient’ n = 80; ‘The mysterious brain’ n = 84; ‘Until death do us part’ n = 39).

### Qualitative analysis of students’ reflections

#### Reflection on a professional level

Students reflected on their role as a healthcare professional (HCP). They reflected on their current role as clinical interns and linked the lesson material to their own experiences in their internships. For example, one student *(quote 1, *Table 2*)* reflected on her own actions in the clinic and reported being more aware of the consequences of these actions after completing the NM assignment.

**Table 2:** Thematic analysis of students’ essays

Theme	Subtheme	Representative quotation
Reflection on a professional level	Current role as HCP	1) “The film and the book made me reflect on how I treat people and what the effects can be of what I say to them or of medical tests I make them undergo.”* (DP46)*
	Future intentions as HCP	2) “No matter how long you are a doctor and how many times you’ve seen the same disease, don’t lose your empathy for your patients.” *(B35)* 3) “When communicating with an autistic patient it’s very important to be in a low-stimulus environment and to deliver an unambiguous message.” *(B41)* 4) “Recognize that patients are people and look beyond just the treatment you can offer, and also look at the bigger picture. Treatment isn’t always the best way to deliver health care, but it’s also important to create understanding and to build a good relationship with your patient.” *(DP21)*
Reflection on a personal level	Life lessons	5) “From the book I take away that there’s more in life than being a doctor and that you need to realize that life can go by quickly. I don’t need 80-h work weeks and I would also value having a life outside of the hospital.” *(DP57)* 6) “Psychiatric patients are people too, with their own lived experiences and norms and values.” *(B24)*
Attention to illness experience and individual patient story	More understanding of illness experience	7) “I’ve gained more insight into the surrender that’s expected of patients. At the start of the treatment process, the process is often very unclear for a ‘layman’; they have to take a journey that is unfamiliar to their current world.” *(DP13)*
	Importance of patient story	8) “Empathizing with a patient is essential to offering optimal care. As each patient is different, perceives the world differently and therefore has different needs, we as doctors need to be sharp and alert to these differences during each consultation.” *(B10)*
	Awareness of gap between doctor and patient	9) “What I got from this exercise is that it’s very important to keep empathizing with the patient as far as that’s possible, because a patient has a different vision and also finds different things important in illness than you as a doctor.” *(DP75)*
Topics related to specific book/film pairing	Awareness of mortality of a doctor	10) “I notice frequently with diseases that I think to myself, I’m not going to get them, and that I consider myself a very healthy person. Still, I’m as equally likely as any other human to get sick.” *(DP67)*
	Insight into specific illness	11) “I’ve learned to better understand how a brain of someone with autism works: Many stimuli come in, which makes it sometimes seem like someone has limited attention (for example, not paying attention to the traffic when crossing a street).” *(B41)*
	Role of the system around a patient	12) “In general, the original treatment stops when the patient leaves the hospital. However, other people continues to provide care at home (caregiver/home care). Also think about how a disease/diagnosis affects the people around a patient.”* (D34)*

*HCP *Health-care professional*, DP *The doctor as a patient, *B* The mysterious brain, *D* Until death do us part

Students also set intentions for their role as a future HCP. Many of these students linked a lesson they had learned from the exercise to what they considered ‘good healthcare’ in general or to what they intended to include into their future practice *(quote 2, *Table 3*)*. Other students articulated more specific intentions, like communication strategies they intended to use in future interactions with (similar) patients *(quote 3, *Table 2*)* or possible care options for patients similar to the ones depicted in the art forms. Students also described the importance of considering more than treatment options alone *(quote 4, *Table 2*)*, and some students even connected these intentions to shared decision-making practices.

**Table 3:** Reflective quality of students’ essays

Score	Criteria based on the REFLECT rubric [28]	Number of essays	Representative reflection
1. Habitual action	- Superficial and descriptive writing. No reflection or introspection - No analysis or meaning making - No authenticity in writing	43 (21,4%)	“I’ve learned what the effect of a disease can be on people around a patient. Empathize with family/people around a patient.” *(D25)*
2. Thoughtful action or introspection	- Elaborated descriptive writing. No reflection - No/little analysis or meaning making - No/little authenticity in writing	58 (28,8%)	“Not a simple image of autism, the different forms of autism require a different approach. It is important to emphasize with the patient to come to shared-decision making. This way, you will understand certain considerations in a better way.” *(B1)*
3. Reflection	- Descriptive as well as reflective writing - Some analysis and meaning making - Writing is somewhat authentic	64 (31,8%)	“I thought that the book and the film both described the finiteness of life in terms of becoming ill, even though the doctor [in the story] was cured he was still really ill. You cannot plan this beforehand, so enjoy and be grateful for all the possibilities that you get in life, because they can suddenly be taken we from you. After all, doctors can also get sick.” *(DP28)*
4. Critical reflection	- Deeper reflection: exploration and questioning of own perspective - Comprehensive analysis and meaning making - Writing is authentic	36 (17,9%)	“In the book, the question is raised “What makes life meaningful enough to go on living” (p. 71). I think this is a very good question that reaches beyond being ‘medically’ healthy. The question made me think about what’s really important in a worthwhile life. Is it well-being? Is it the capability to communicate? Language? These are questions Kalanithi thought about, too. Today we’ve also learned that people with a handicap are sometimes happier than ‘healthy’ people. I’ll take the question from the book with me to continue to reflect upon and as a basis for my choices in the future.” *(DP76)*

*DP *The doctor as a patient, *B* The mysterious brain, *D* Until death do us part

#### Reflection on a personal level

Students described lessons they had learned that had an impact on their personal worldview or beliefs. They reflected on health, illness and death and connected this to their opinion of a ‘meaningful life’. They thought about what made them happy and mentioned the importance of finding a balance between work and private life *(quote 5, *Table 2*).* In addition, some students reported gaining more insight into their own biases and stereotypes, for example about psychiatric patients *(quote 6, *Table 2*).*

#### Attention to illness experience and individual patient story

Students mentioned that the stories in the book and film pairings helped them understand more about illness experiences or perspectives of patients. They expressed gaining more insight into the problems or dilemmas patients face. For example, a student *(quote 7, *Table 2*)* mentioned a new awareness of a patient’s vulnerability. Many students also noticed the individual nature of each patient’s story and life experiences, resulting in different views, wishes, and beliefs. They also mentioned how important it is to be aware of this as a HCP *(quote 8, *Table 2*),* and frequently linked this to future intentions*.* Furthermore, some students reported an awareness of how their own life story and experiences influenced their worldview, which they described as a gap between themselves and their patients *(quote 9, *Table 2*).* Some also expressed awareness of their own limitations in understanding people’s feelings.

#### Topics related to specific book/film pairings

The themes mentioned above were found in all three book and film pairings. We also found themes which were related to specific book and film combinations. In the pairing ‘The doctor as a patient’, some students expressed a new awareness of the possibility of getting sick or dying as a HCP themselves. As illustrated by one student *(quote 10, *Table 2*)*, they expressed the distance they normally felt between their own health and that of the patients they worked with every day. For the pairing ‘The mysterious brain’, students mentioned gaining more insight into a specific disease—autism spectrum—as well as the workings of an autistic brain *(quote 11, *Table 2*)*. This new knowledge, according to their reflections, provided a new perspective to what they had learned in the standard curriculum. In the third pairing, ‘Till death do us part’, students expressed more understanding of the role of the support system around the patient. As one student wrote *(quote 12, *Table 2*)*, the art forms illuminated how burdensome it could be for family members and loved ones to care for someone ill. We also found this theme in ‘The mysterious brain’, where students mentioned the importance of a good support system for optimal treatment outcomes.

### Quantitative analysis of students’ level of reflection

Table 3 shows the distribution of reflection levels and representatives quotes. Approximately half of the students showed an in-depth reflection (score of 3 or 4) These students made a connection between what they read or saw and their own experiences, values, or beliefs. They demonstrated a process of analysis and meaning making. Their reflections furthermore displayed a distinct and authentic writerly voice. Some of these students also demonstrated critical reflective skills by questioning their own norms and values and by trying to articulate a deeper understanding of the dilemmas illustrated in the book and film.

The other half of the students used more descriptive writing in their essays (score of 1 or 2). In such essays, students only showed habitual actions, meaning they described general statements and abstract lessons. Others wrote more elaborative descriptions, but they still didn’t connect this to their own experiences. These reflections remained superficial as there was little or no meaning-making. Also, in many of these essays, there was a lack of an authentic or distinct writerly voice.

The median level of reflection seen in the students’ essays was 2 (*n* = 203; IQR 2-3). The reflection level differed between the book and film pairings (*p* < 0.01), with a mean score of 3 (*n* = 79; IQR 2-4) for ‘The doctor as a patient’, 2 (*n* = 83; IQR 1-3) for ‘The mysterious brain’ and 2 (*n* = 39; IQR = 1-2) for ‘Till death do us part’.

### Students’ experience of the NM lesson

Students appreciated the artforms and used terms like ‘enjoyable’ and ‘interesting’ to describe the assignment *(quote 1, *Table 4*).* Learning about illness in a different way gave them new insights in comparison to the standard curriculum *(quote 2, *Table 4*).* Students also mentioned that the discussion with their fellow students enriched the learning process *(quote 3, *Table 4*).* Furthermore, a number of students mentioned the supportive classroom atmosphere, a combination of the small group setting and the guidance of the teacher *(quote 4, *Table 4*).*

**Table 4:** Thematic analysis of students’ experience of lesson

Theme	Representative quote
Enjoyed art forms	1) “Substantively enjoyable, interesting and relevant book and film.” *(B42)*
Novel approach to medical topic	2) “Nice assignment, good way to broaden our medical education (and literature) and to think about ethical issues. Less reproduction than the rest of the study, rather more critical thinking, discussing and questioning.” (DP59)
Learning by discussing with peers	3) “The conversation […] led to more in-depth questioning, and it was interesting to hear what other students thought.” *(DP26)*
Supportive classroom atmosphere	4) “Very pleasant and safe atmosphere in the lesson, where you’re free to tell everything and be open about your opinion or feelings.” *(DP21)*
Suggestions for improvement	5) “More freedom of choice in the book/film combinations, let us choose from different options. Better availability of book/film.” *(D21)*

*DP* The doctor as a patient, *B* The mysterious brain, *D* Until death do us part

Most of the suggestions for improvement pertained to practical issues related to scheduling and the availability of the lesson materials *(quote 5, *Table 4*).* Some students were critical to the choice of the art forms, especially in the pairing ‘Till death do us part’. A few students considered the time investment of the assignment too great.

## Discussion

This study showed that a mandatory narrative medicine intervention, where students read a book and watched a film, led to reflection on themes related to patient-centeredness in a large study sample of medical students.

The role of narrative medicine in the medical curriculum has been studied in the past, and the themes we found in this research correspond with other findings on this subject, namely participant satisfaction, perspective taking and self-reflection [14–16]. However, most of these studies included a small study population, predominantly in elective courses. We were able to validate these positive results in a larger population. More importantly, we’ve explored a mandatory narrative medicine lesson for all fourth-year medical students and not just for students with a predisposition for this field. Furthermore, this is the first study that combined a book and film in a narrative medicine exercise. By using two different media we aimed to interest students who might feel some resistance toward reading, and more broadly, to narrative medicine activities. As Arntfield et al. [17] mention, humanities-based programs are sometimes viewed as ‘counter-culture’, especially by those unfamiliar with the field. Also, by using two different media with the same topic, we encouraged students to think about similarities and differences between these two perspectives. Besides that, we demonstrated that the different book-film pairings taught students overarching lessons as well as lessons specific to the thematic pairings. This can be further optimised in teaching targeted lessons throughout the medical curriculum.

The content of students’ reflections aligns with underlying principles of patient-centered care as described by Scholl et al. [4, 5]: “the clinician-patient relationship”, “patient as a unique person” and “essential characteristics of the clinician”. Regarding the clinician-patient relationship, some students mentioned communication strategies they could use to create a deeper connection with their patients in general and with specific patient groups. Others gained more insight into their own attitudes and how this could influence the relationship. In addition, several students gained more awareness of the individual illness experience, in other words, the unique nature of each patient’s story. They mentioned the individual perspectives and priorities of patients and how personalised treatment can complement these values. Various students also demonstrated relevant clinician characteristics related to patient-centeredness. They showed empathy and compassion towards the characters depicted in the artforms and were able to connect these narratives to the patients they encountered in their clinical internships.

Additionally, this narrative exercise stimulated meaningful self-reflection; another clinician characteristic related to patient-centered care[4, 5]. Even though this was only a short reflective writing exercise, approximately half of the students showed an in-depth reflection. This is probably an underestimation of the amount of students who actually reached in-depth reflection, but did not write this down during the brief writing exercise. Furthermore, a major part of the self-reflection took place during the group discussion itself. The choice of book and film pairing also influenced the level of reflection in the essays. Students who were assigned to the pairing ‘The doctor as a patient’ showed more reflection compared to students who were assigned to other pairings. We hypothise that the theme ‘The doctor as a patient’ related most to students’ personal and professional lives. A perceived affinity with the characters in the artforms can be correlated with more identification with these characters. This process of identification is known to influence the beliefs and values of the readers [30].

Other factors might also have played a role in the richness of the essays. For instance, it is known that teacher qualities can influence students’ learning motivations and academic performance [31]. Variations in teachers’ explanation of the writing exercise as well as their verbal and non-verbal communication during the lesson might have influenced students’ reflections and observations. This is a topic that deserves future research.

While this study showed positive effects on students’ patient-centeredness, we do not know to what extent this lesson ultimately impacts students’ reflective capacity and patient-centeredness in the clinical setting. This is due to the study design and the short nature of the NM intervention. Also, faculty members were free to change elements of their lessons to their own liking. As a result, we noticed variations in length and structure of the writing exercises; some faculty members didn’t even ask students to write down their reflections. A significant amount of students were excluded from this study because their essays were illegible, they didn’t complete the assignment, or their teachers didn’t submit the essays them properly. These teacher and student factors could have led to a positive selection bias of the results. Because it was a short, guided reflection, some students might have given the obvious or ‘desired’ answers based on other medical school lecture topics about shared decision-making and patient participation [12]. However, the analysis of students’ written reflections revealed a variety of topics and themes that were broader than the guiding questions.

## Conclusion

In conclusion, this narrative medicine lesson at the UMCU facilitated reflection on multiple aspects of patient-centeredness among fourth-year medical students. This research underlines the value of narrative medicine in the standard medical curriculum by validating various purported outcomes (for instance, contributing to patient-centeredness) in a larger study population and a mandatory course. Going forward, NM exercises should be more thoroughly integrated in the medical curricula to provide students with more continuity during their education and to teach them long-term skills for their future careers. Future research should focus on how students transfer these skills into clinical practice and how this can be optimized.

## Data Availability

The datasets generated and analysed during the current study are not publicly available due to participant privacy reasons, but are available from the corresponding author on reasonable request.

## Abbreviations

HCP: Healthcare professional
NM: Narrative medicine
NVMO: Netherlands Association of Medical Education
REFLECT: The Reflection Evaluation For Learners’ Enhanced Compentencies Tool
UMCU: University Medical Center Utrecht
